# Factors Associated with Progression of Atrial Fibrillation and Impact on All-Cause Mortality in a Cohort of European Patients

**DOI:** 10.3390/jcm12030768

**Published:** 2023-01-18

**Authors:** Marco Vitolo, Marco Proietti, Jacopo F. Imberti, Niccolò Bonini, Giulio Francesco Romiti, Davide A. Mei, Vincenzo L. Malavasi, Igor Diemberger, Laurent Fauchier, Francisco Marin, Michael Nabauer, Tatjana S. Potpara, Gheorghe-Andrei Dan, Gregory Y. H. Lip, Giuseppe Boriani

**Affiliations:** 1 Cardiology Division, Department of Biomedical, Metabolic and Neural Sciences, University of Modena and Reggio Emilia, Policlinico di Modena, 41124 Modena, Italy; 2 Liverpool Centre for Cardiovascular Science at University of Liverpool, Liverpool John Moores University, Liverpool Heart & Chest Hospital, Liverpool L14 3PE, UK; 3 Clinical and Experimental Medicine PhD Program, University of Modena and Reggio Emilia, 41125 Modena, Italy; 4 Department of Clinical Sciences and Community Health, University of Milan, 20122 Milan, Italy; 5 Geriatric Unit, IRCCS Istituti Clinici Scientifici Maugeri, 20138 Milan, Italy; 6 Department of Translational and Precision Medicine, Sapienza—University of Rome, 00185 Rome, Italy; 7 Department of Experimental, Diagnostic and Specialty Medicine, Institute of Cardiology, University of Bologna, Policlinico S. Orsola-Malpighi, 40138 Bologna, Italy; 8 Service de Cardiologie, Centre Hospitalier Universitaire Trousseau, 37000 Tours, France; 9 Department of Cardiology, Hospital Universitario Virgen de la Arrixaca, IMIB-Arrixaca, University of Murcia, CIBERCV, 30100 Murcia, Spain; 10 Department of Cardiology, Ludwig-Maximilians-University, 80539 Munich, Germany; 11 School of Medicine, University of Belgrade, 11000 Belgrade, Serbia; 12 Intensive Arrhythmia Care, Cardiology Clinic, Clinical Center of Serbia, 11000 Belgrade, Serbia; 13 ‘Carol Davila’ University of Medicine, Colentina University Hospital, 020125 Bucharest, Romania; 14 Department of Clinical Medicine, Aalborg University, 9220 Aalborg, Denmark

**Keywords:** atrial fibrillation, atrial fibrillation type, remodeling, progression, outcomes, death, registry

## Abstract

Background: Paroxysmal atrial fibrillation (AF) may often progress towards more sustained forms of the arrhythmia, but further research is needed on the factors associated with this clinical course. Methods: We analyzed patients enrolled in a prospective cohort study of AF patients. Patients with paroxysmal AF at baseline or first-detected AF (with successful cardioversion) were included. According to rhythm status at 1 year, patients were stratified into: (i) No AF progression and (ii) AF progression. All-cause death was the primary outcome. Results: A total of 2688 patients were included (median age 67 years, interquartile range 60–75, females 44.7%). At 1-year of follow-up, 2094 (77.9%) patients showed no AF progression, while 594 (22.1%) developed persistent or permanent AF. On multivariable logistic regression analysis, no physical activity (odds ratio [OR] 1.35, 95% CI 1.02–1.78), valvular heart disease (OR 1.63, 95% CI 1.23–2.15), left atrial diameter (OR 1.03, 95% CI 1.01–1.05), or left ventricular ejection fraction (OR 0.98, 95% CI 0.97–1.00) were independently associated with AF progression at 1 year. After the assessment at 1 year, the patients were followed for an extended follow-up of 371 days, and those with AF progression were independently associated with a higher risk for all-cause death (adjusted hazard ratio 1.77, 95% CI 1.09–2.89) compared to no-AF-progression patients. Conclusions: In a contemporary cohort of AF patients, a substantial proportion of patients presenting with paroxysmal or first-detected AF showed progression of the AF pattern within 1 year, and clinical factors related to cardiac remodeling were associated with progression. AF progression was associated with an increased risk of all-cause mortality.

## 1. Introduction

Atrial fibrillation (AF) is a dynamic disease and may evolve over time [1]. Several classifications or characterizations of AF have been proposed [2,3], but in clinical practice AF is classified into five patterns according to presentation, duration, and termination of the arrhythmic episodes (i.e., first-diagnosed, paroxysmal, persistent, long-standing, persistent, and permanent) [4]. Paroxysmal AF often shows a natural progression towards more sustained forms of the arrhythmia. From a pathophysiological point of view, aging, cardiovascular (CV) risk factors, and comorbidities are associated with atrial remodeling, promoting the transition from paroxysmal to non-paroxysmal AF [3,5,6]. However, the true incidence of AF progression has not been well-defined and has greatly varied among previous studies depending on the exact definition of arrhythmia progression, characteristics of the population included, follow-up, and type of monitoring [7].

Previous studies showed that progression to a more sustained form of AF may also be associated with adverse CV outcomes and all-cause mortality [8]. For these reasons, identifying patients at risk of progression is essential to possibly reduce and slow down the rate of AF progression with the aim of improving patients’ outcomes.

In the present analysis from a contemporary European multicentre cohort of AF patients, we aimed to investigate clinical factors associated with progression of AF and its impact on adverse outcomes.

## 2. Methods

### 2.1. Study Design and Cohort

The present analysis is derived from a prospective, observational, large-scale multicentre study of AF patients. A complete description of the study design, inclusion and exclusion criteria, baseline characteristics, and follow-up results has been reported elsewhere [9,10,11]. In brief, the registry enrolled consecutive AF patients (both in- and out-patients) in 250 centres across 27 participating countries from October 2013 to September 2016. All the patients were aged ≥ 18 years old, provided written informed consent, and had documented AF within 12 months before enrolment. An institutional review board approved the study protocol for every institution. The study was performed according to the EU Note for Guidance on Good Clinical Practice CPMP/ECH/135/95 and the Declaration of Helsinki.

Thromboembolic and bleeding risk were defined according to CHA_2_DS_2_-VASc score [12] and HAS-BLED score [13], respectively. The severity of AF-related symptoms was defined according to EHRA score [4].

The type of AF was classified according to European Guidelines [14] (i.e., first-detected AF, paroxysmal AF, persistent AF, long-standing persistent AF, and permanent AF) and was defined by the investigator at baseline and at 1 year of follow-up.

For the purpose of this analysis, only patients with paroxysmal AF at baseline or first- detected AF in whom sinus rhythm was restored spontaneously or after successful cardioversion (either pharmacological or electrical) were included. Patients with persistent, long-standing persistent, or permanent AF at baseline; unknown rhythm status at baseline and/or at 1-year of follow-up; or no follow-up data available were excluded.

### 2.2. Atrial Fibrillation Progression

Patients with known rhythm status at 1 year were stratified into two groups: (i) No AF progression and (ii) AF progression. Progression of AF was defined as follows: paroxysmal AF at baseline or first-detected AF underwent successful cardioversion during admission/consultation at baseline, becoming persistent or permanent AF at 1-year follow-up as per adjudication by the investigators. In the present study, patients with persistent AF and long-standing persistent AF were reported together. We also tested the association between the application of a rhythm-control strategy at baseline and progression of AF. Rhythm-control strategies included, alone or in combination, electrical cardioversion, pharmacological cardioversion, catheter ablation, and the use of antiarrhythmic drugs (AADs, Class Ia, Class Ic, Class III).

### 2.3. Follow-Up and Adverse Outcomes

For the present analysis, incident major adverse clinical events were evaluated starting from the first year of follow-up after the assessment of AF progression. The following adverse events were reported according to the two groups (i.e., AF progression vs No AF progression): (i) all-cause death; (ii) CV death; (iii) any thromboembolism (TE) (including stroke, transient ischaemic attack [TIA], and any peripheral embolism); (iv) any ACS; (v) major bleedings; and (vi) hospitalization for heart failure. All-cause death was the primary endpoint of the present analysis. The composite outcome of any TE/any ACS/CV-death, defined as Major Adverse Cardiovascular Events (MACE), was also evaluated.

### 2.4. Statistical Analysis

All continuous variables were described as median and interquartile range (IQR). The Mann-Whitney U or Kruskal-Wallis tests were used to perform among-group comparisons, where appropriate. Categorical variables were reported as counts and percentages. Among-group comparisons were made using a χ^2^ test or Fisher’s exact test (if any expected cell count was less than five).

Univariable and multivariable logistic regression analyses were performed to identify baseline characteristics associated with AF progression at 1 year of follow-up. All variables with *p* < 0.10 in the univariable analysis were used in the multivariable model to identify independent clinical factors associated with AF progression. The association between rhythm-control interventions at baseline and AF progression at 1 year was assessed by using two different multivariable models: Model 1 was adjusted for age, whereas Model 2 was adjusted for the CHA_2_DS_2_VASc score. The results were expressed as odds ratio (OR) and 95% confidence interval (CI).

After the assessment of AF progression at 1-year, adverse events were collected at 2 years of follow-up. Plots of Kaplan-Meier curves for time to all-cause death according to AF progression assessed were performed. Survival distributions were compared using the log-rank test.

Cox regression analysis was used to establish the relationship between AF progression and the risk of adverse outcomes. For the primary endpoint of all-cause death, the analysis was further adjusted for age, sex, heart failure, coronary artery disease, hypertension, diabetes mellitus, previous TE, peripheral artery disease, chronic kidney disease, and use of oral anticoagulants. The results were expressed as hazard ratio (HR) and 95% confidence interval (CI).

A two-sided *p*-value < 0.05 was considered statistically significant. All the analyses were performed using SPSS statistical software (version 26.0, Statistical Package for the Social Sciences, SPSS, IBM, Chicago, IL, USA).

## 3. Results

**Figure 1:**
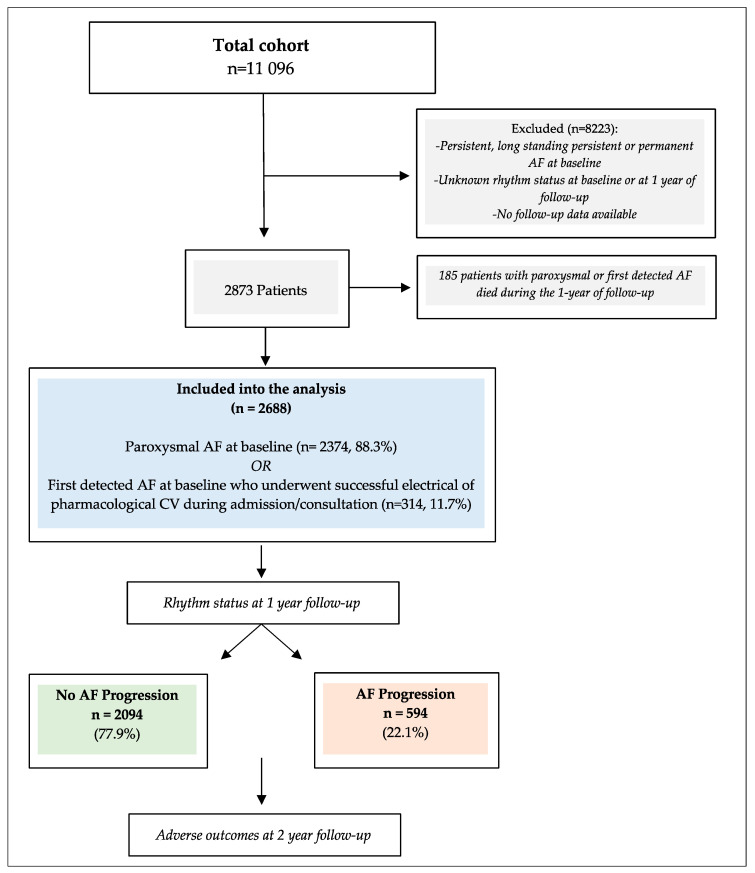
Study flow diagram. AF = atrial fibrillation; CV = cardioversion.

Among the 11096 AF patients originally enrolled in the Registry, a total of 2688 patients with paroxysmal AF at baseline or first-detected AF (with successful cardioversion) and available data on AF progression at 1 year of follow-up were included in the present study (Figure 1). The median age was 67 years [IQR 60–75], with a higher proportion of male patients (55.3%). The median [IQR] CHA_2_DS_2_VASc and HASBLED scores were 3 [1,2,3,4] and 1 [1,2], respectively.

### 3.1. Atrial Fibrillation Progression

At 1 year of follow-up, 2094 (77.9%) patients showed no AF progression, while 594 (22.1%) developed persistent or permanent AF. Baseline characteristics stratified by AF progression at 1 year are shown in Table 1. Patients with AF progression were older with a higher prevalence of comorbidities such as hypertension, heart failure, and valvular heart disease, and a higher CHA_2_DS_2_VASc score compared to patients who did not show arrhythmia progression (Table 1). Pharmacological management and antithrombotic treatment are shown in Supplementary Materials (Table S1). Patients with AF progression were more frequently treated with a higher number of concomitant medications compared to patients without arrhythmia progression. Polypharmacy (i.e., contemporary use of five or more drugs) was more prevalent in patients with arrhythmia progression (51.9% vs. 45.3%, *p* = 0.005) (Table S1). However, some differences regarding the specific type of pharmacological treatment used were evident. For example, patients without AF progression were more frequently treated with class IC antiarrhythmic drugs such as flecainide or propafenone; conversely, amiodarone, digoxin, aldosterone blockers, and diuretics were more prescribed in patients with AF progression, probably reflecting the higher prevalence of heart failure and CV comorbidities in this group (Table S1).

### 3.2. Clinical Factors Associated with AF Progression and Rhythm-Control Interventions

Table 2 shows the univariable and multivariable logistic regression analysis for clinical factors associated with AF progression at 1 year of follow-up. In the multivariable logistic regression analysis, no physical activity (OR 1.35, 95% CI 1.02–1.78), valvular heart disease (OR 1.63, 95% CI 1.23–2.15), left atrium diameter (OR 1.03, 95% CI 1.01–1.05), and left ventricular ejection fraction (OR 0.98, 95% CI 0.97–1.00) were independently associated with AF progression at 1 year.

Overall, there were no differences in rhythm-control interventions between patients with and without AF progression (Table 3). Additionally, patients treated with or without rhythm-control strategy had similar rates of AF progression (21.5% vs. 23.0%, *p* = 0.32). On univariable and multivariable analysis, there was no significant association between the use of rhythm-control strategies and AF progression (Table 3). Some differences regarding the specific type of rhythm-control option and progression of AF were evident. Compared to the use of only AADs, catheter ablation was inversely associated with AF progression on univariable analysis (OR 0.43, 95% CI 0.20–0.92). A similar trend was found even after the adjustments for age and CHA_2_DS_2_VASc score (Table 3). Conversely, a rhythm-control strategy including only the use of cardioversion (either electrical or pharmacological) was independently associated with AF progression (Table 3).

### 3.3. Follow-Up and Adverse Outcomes

**Figure 2:**
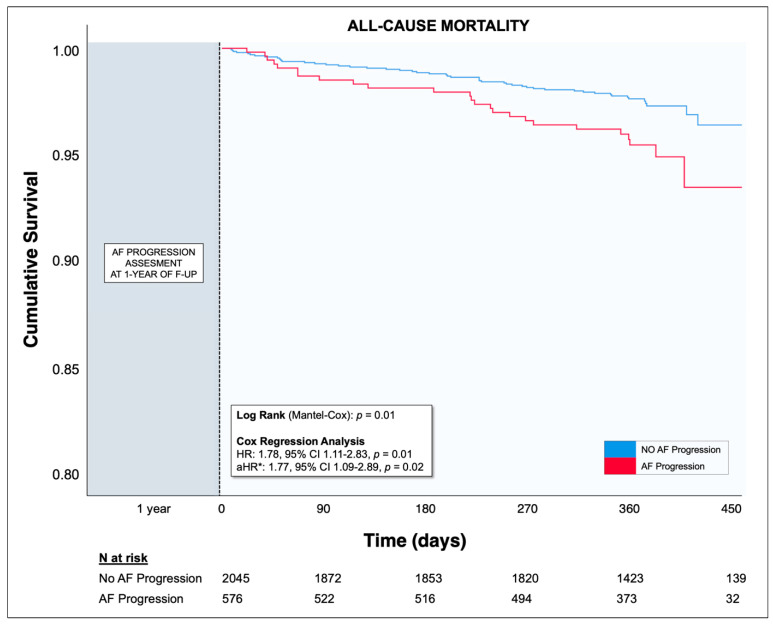
Kaplan-Meier curves for the primary endpoint (all-cause death) for patients with or without AF progression during the extended follow-up (i.e., after AF progression assessment at 1-year). aHR = adjusted hazard ratio; AF = atrial fibrillation; CI confidence interval; F-UP = follow-up; HR, hazard ratio; N = number. * The Cox regression analysis was adjusted for age, sex, heart failure, coronary artery disease, hypertension, diabetes mellitus, previous thromboembolic events, peripheral artery disease, chronic kidney disease, and use of oral anticoagulants.

Major adverse events following the assessment of AF progression are shown in Table 4. After a median follow-up of 371 [IQR 345–388] days starting from 1 year, there were 80 (3.1%) deaths with a significantly lower proportion in patients without AF progression (2.6% vs. 4.5%, *p* = 0.02). Kaplan-Meier analysis showed a lower cumulative survival of patients with AF progression compared to patients with no AF progression (Log Rank *p* = 0.01) (Figure 2). On multivariable Cox regression analysis, adjusted for age, sex, heart failure, coronary artery disease, hypertension, diabetes mellitus, previous TE, peripheral artery disease, chronic kidney disease, and use of oral anticoagulants, patients with AF progression had an independently higher risk for all-cause death (adjusted HR 1.77, 95% CI 1.09–2.89) compared to patients without arrhythmia progression. For the other outcomes of interest, no significant differences were found between patients with and without AF progression (Table 4).

## 4. Discussion

The principal findings of this analysis based on a large cohort of AF patients are as follows: (i) a substantial proportion of patients (around 22%) with paroxysmal AF or newly detected AF successfully cardioverted at baseline, progressed to a more sustained form of arrhythmia at 1 year of follow-up; (ii) clinical factors related to cardiac structural remodeling were independently associated with arrhythmia progression; and (iii) AF progression was independently associated with all-cause mortality during the subsequent follow up.

Atrial fibrillation is a dynamic disease, and its natural history commonly shows a natural progression from paroxysmal to more sustained forms. The progressive nature of AF episodes led to the well-known concept of “AF begets AF” [15]. Previous studies evaluating the rate of progression in AF in the general population have reported conflicting results, with progression rates ranging from 2% to 20% per patient-year [16,17]. The discrepancies in the reported progression rates depend on the characteristics of the cohorts analyzed, the duration of follow-up, the type of monitoring, and the definition of AF progression itself.

Our study found that around 22% of real-world AF patients showed arrhythmia progression (to either persistent or permanent AF) at 1 year, which corresponds to the upper limit of previously reported data on this topic. However, most of the previous studies included only patients with paroxysmal AF at baseline [17]. One of the strengths of our analysis lies in the fact that we also included patients with newly detected AF treated with successful cardioversion (almost 11%), thus providing new insights in a real-world population not fully evaluated in terms of arrhythmia progression.

Other observational data need to be interpreted according to the different time periods in which variable approaches for rhythm control have been used. In the Euro Heart Survey performed in the years 2003–2004 and including 1219 patients [18], progression of AF to more sustained forms occurred in 15% of patients at 1 year. The extended follow-up analysis of the Canadian Registry of Atrial Fibrillation (CARAF) study enrolled in the 1990s showed that after a median follow-up of 6.35 years, the rate of progression from paroxysmal to persistent AF at 1, 5, and 10 years was 8.6%, 24.3%, and 36.3%, respectively [19]. Recent data from the AF-RISK study interestingly found higher AF progression rates in persistent (26%), compared to paroxysmal AF (11%) at 1 year [5]. A recent meta-analysis including more than 27,000 patients showed that the pooled incidence of AF progression was 8.1% per patient-year of follow-up [17].

Beyond these epidemiological considerations, the clinical challenge is identifying patients at risk for AF progression with the aim of preventing or delaying the natural course of the arrhythmia. Several cardiovascular risk factors and comorbidities may promote structural and functional modifications of the atria, acting as a favorable substrate for the development, maintenance, and progression of AF [20,21]. In our analysis, different clinical factors which may be related to cardiac remodeling, such as valvular heart disease, heart failure, or LA diameter, were independently associated with AF progression. Previously, different prediction schemes for progression of AF have been identified, based on similar clinical characteristics as we found in our cohort. For example, the HATCH score (hypertension, age ≥ 75 years, stroke or TIA, chronic obstructive pulmonary disease, and heart failure) was specifically validated to predict the likelihood of AF progression, showing a good predictive ability (area under the curve = 0.675, 95% CI 0.632–0.718) [18]. In a recent analysis of unselected general AF patients [22], both CHA_2_DS_2_VASc and HATCH scores were incrementally associated with progression to permanent AF, highlighting the contributions of common CV risk factors or comorbidities on the arrhythmia progression. Interestingly, adding LA dilation (moderate–severe volume increase) to these clinical scores improved the prediction of progression to permanent AF [22].

Differently from our study, previous analyses have identified age as a common risk factor for AF progression [17,23]. Advancing age may promote atrial structural remodeling through different pathophysiological mechanisms [1,17]. In our analysis, age was a predictor of AF progression only on univariable analysis, suggesting that increasing age may be a risk factor only if it is associated with other comorbidities acting as risk modifier. In the Fushimi AF registry, for example, factors included in the HATCH score were not independent predictors of AF progression and the interval, since the first detection of AF rather than age was a risk factor for progression [24].

Beyond the above-mentioned associations between CV risk factors/comorbidities and AF progression, our results interestingly suggest that also the degree of physical activity modulates the occurrence of disease progression. In our study, among the patients with AF reporting no physical activity, there was indeed almost 35% increased odds of AF progression. This is in line with prior studies showing lack of physical activity being related to AF incidence [25]. However, the association between AF and physical activity has been an object of debate for several years, and previous studies reported sometimes conflicting results [26,27]. Some studies indeed found a U-shaped relationship between physical activity and AF incidence and progression, so our study, despite its observational nature, may contribute to additional knowledge on the benefits of physical activity [26,28].

Indeed, our findings extend what was found in a previous analysis on the EORP General Registry where the occurrence of AF progression during a 1-year follow up in patients reporting no physical activity appeared numerically higher, as compared to patients with intense physical activity (17.7% vs. 6.8%), although without achieving statistical significance [29].

### 4.1. AF Progression and the Risk of Outcome 

The main finding of our analysis was that progression from paroxysmal to persistent or permanent AF resulted in an independent increased risk of all-cause death. Of note, this effect was observed even after the adjustments for several confounders including the individual components of the CHA_2_DS_2_-VASc score, chronic kidney disease, and use of oral anticoagulants, which are known determinants of all-cause death in the AF population.

Previous studies highlighted that AF progression may be associated with major adverse cardiovascular outcomes such as myocardial infarction, thromboembolism, heart failure, stroke, and all-cause death [5,30]. In the AF-Risk study, which was based on an advanced type of monitoring, not applicable to the real world (repeated Holter monitoring over 1 year), patients with AF progression had more CV events and all-cause mortality [5]. The Fushimi AF Registry, collecting patients from Japan, found that progression of AF was associated with an increased risk of ischemic stroke or systemic embolism and hospitalization for heart failure. We could presume that the amount of AF itself, expressed as AF burden, could have prognostic implications. However, in daily practice it is difficult to precisely evaluate the association between AF burden and adverse outcomes. Therefore, the definition of progression of AF has inherent methodological limitations, and more precise assessments could only be performed by studies performed in the specific setting of patients with cardiac electronic implantable devices. [31]. Recent evidence also suggests a dose-response relationship between AF burden and the risk of stroke (despite this risk being nonlinear) [32,33,34].

Taken together, these results reinforce the concept that delay of progression may be an important measure to limit the adverse outcomes of AF. Contemporary management of AF is based on the three pillars of the ABC pathway (i.e., anticoagulation, better symptom management, and cardiovascular and other comorbidities management including lifestyle changes) [2,35,36,37]. Beyond the indisputable benefits of anticoagulation treatment in patients at risk of stroke, we are seeing a paradigm shift in the treatment of AF patients, particularly in terms of rhythm control [38,39]. Recent studies have reported that early rhythm control rather than rate control for selected patients with new-onset AF, together with appropriate anticoagulation treatment, may be associated with improved outcomes [40]. Early use of rhythm-control management could indeed reduce irreversible atrial remodeling, delaying progression of AF with the final aim of improving clinical outcomes [41,42,43]. The role of rhythm-control interventions in the prevention of AF progression is difficult to interpret and may be conditioned by possible selection biases. In our study, we found that overall, AF patients treated with and without rhythm-control strategies had a similar rate of AF progression at 1 year of follow-up but that the use of catheter ablation tended to be inversely associated with progression. Whilst data from observational studies should be interpreted with caution, both our results and the results of two large prospective cohort studies based in Switzerland similarly showed that rhythm-control interventions had no effect on AF progression, while pulmonary vein isolation was associated with a lower degree of AF progression [17]. Of note, the recent EARLY AF Trial found that early treatment of AF patients with catheter cryoballoon ablation was associated with a lower incidence of persistent AF or recurrent atrial tachyarrhythmia over 3 years of follow-up than initial use of antiarrhythmic drugs [44].

### 4.2. Study Limitations

Our study has inherent limitations that should be acknowledged. The main limitation of our study is related to its observational nature and to the study setting which is based exclusively on cardiology practices. A specific limitation of our analysis, based on real-world practice, relies on the definition of AF progression, based on a clinical assessment and not on specific tools for continuous rhythm monitoring. Additionally, specific data on type of ablation performed are lacking, thus limiting a granular analysis on this issue. Given the relatively small sample size and the number of events, our study should be considered as hypothesis-generating, reporting associations but not implying causality.

## 5. Conclusions

In a contemporary cohort of AF patients, a substantial number of patients (around 22%) progressed to sustained AF within 1 year, and clinical factors related to cardiac structural remodeling were associated with progression. This latter was associated with an increased risk of all-cause mortality.

## Figures and Tables

**Table 1:** Baseline characteristics.

	No AF Progression (n = 2094, 77.9%)	AF Progression (n = 594, 22.1%)	Total (n = 2688)	*p*
Age (years), median (IQR)	67 (59–75)	69 (61–76)	67 (60–75)	<0.001
Female, n (%)	952/2094 (45.5)	249/594 (41.9)	1201 (44.7)	0.12
BMI (kg/m^2^), median (IQR)	27.2 (24.6–30.5)	28.0 (25.0–31.5)	27.4 (24.7–30.7)	0.005
Site of inclusion, n (%)				0.15
*Hospital*	1069/2094 (51.1)	323/594 (54.4)	1392/2688 (51.8)	
*Outpatient or office based*	1025/2094 (48.9)	271/594 (45.6)	1296/2688 (48.2)	
Hypertension, n (%)	1192/2085 (57.2)	377/592 (63.7)	1569/2677 (58.6)	0.005
Diabetes mellitus, n (%)	406/2080 (19.5)	132/591 (22.3)	538/2671 (20.1)	0.13
Smoking (current), n (%)	235/1992 (11.8)	58/556 (10.4)	293/2548 (11.5)	0.37
Lipid disorder, n (%)	873/2032 (43.0)	239/563 (42.5)	1112/2595 (42.9)	0.82
No physical activity, n (%)	621/1816 (34.2)	207/504 (41.1)	828/1492 (35.7)	0.004
Heart failure, n (%)	524/2082 (25.2)	202/585 (34.5)	726/2667 (27.2)	<0.001
*NYHA III/IV, n (%)*	113/524 (21.6)	61/202 (30.2)	174/726 (24.0)	0.01
Dilated CMP, n (%)	77/2077 (3.7)	39/588 (6.6)	116/2665 (4.4)	0.002
Hypertrophic CMP, n (%)	43/2079 (2.1)	24/588 (4.1)	67/2667 (2.5)	0.006
PAH, n (%)	63/2074 (3.0)	26/586 (4.4)	89/2660 (3.3)	0.09
Coronary artery disease, n (%)	536/2020 (26.5)	169/567 (29.8)	705/2587 (27.3)	0.12
*Previous MI*	222/536 (41.4)	66/169 (39.1)	288/705 (40.9)	0.58
*Previous PCI*	211/536 (39.4)	71/169 (42.0)	282/705 (40.0)	0.54
*Previous CABG*	76/536 (14.2)	24/169 (14.2)	100/705 (14.2)	0.99
*Previous angina*	217/536 (40.5)	67/169 (39.6)	284/705 (40.3)	0.84
Valvular disease, n (%)	671/2070 (32.4)	268/577 (46.4)	939/2647 (35.5)	<0.001
Previous TE events, n (%)	221/2081 (10.6)	74/589 (12.6)	295/2670 (11.0)	0.18
Previous ischaemic stroke, n (%)	115/2081 (5.5)	32/589 (5.4)	147/2670 (5.5)	0.93
Previous TIA, n (%)	70/2081 (3.4)	26/589 (4.4)	96/2670 (3.6)	0.22
Previous EP/DVT, n (%)	41/2081 (2.0)	18/589 (3.1)	59/2670 (2.2)	0.11
Previous haemorrhagic events, n (%)	86/2079 (4.1)	24/588 (4.1)	110/2667 (4.1)	0.95
Peripheral vascular disease, n (%)	139/2057 (6.8)	41/578 (7.1)	180/2635 (6.8)	0.77
Liver disease, n (%)	39/2084 (1.9)	11/593 (1.9)	50/2677 (1.9)	0.97
COPD, n (%)	116/2075 (5.6)	46/586 (7.8)	162/2661 (6.1)	0.04
Dementia, n (%)	17/2090 (0.8)	6/593 (1.0)	23/2683 (0.9)	0.64
Anaemia, n (%)	74/2090 (3.5)	24/594 (4.0)	98/2684 (3.7)	0.56
Malignancy (current + prior), n (%)	140/2088 (6.7)	46/590 (7.8)	186/2678 (6.9)	0.35
Hyperthyroidism, n (%)	94/2058 (4.6)	32/579 (5.5)	126/2637 (4.8)	0.33
Hypothyroidism, n (%)	216/2061 (10.5)	55/579 (9.5)	271/2640 (10.3)	0.49
CKD, n (%)	185/2086 (8.9)	61/592 (10.3)	246/2678 (9.2)	0.28
CrCl (C-G) (mL/min), median (IQR)	81.6 (60.7–103.9)	73.9 (57.1–97.9)	80.6 (59.9–102.7)	0.007
CHA_2_DS_2_VASc, median (IQR)	3 (1–4)	3 (2–4)	3 (1–4)	<0.001
HASBLED, median (IQR)	1 (1–2)	1 (1–2)	1 (1–2)	0.10
EHRA score, median (IQR)	2 (1–2)	2 (1–2)	2 (1–2)	0.12
*EHRA score I, n (%)*	860/2094 (41.1)	223/594 (37.5)	1083/2688 (40.3)	
*EHRA score II, n (%)*	800/2094 (38.2)	239/594 (40.2)	1039/2688 (38.7)	
*EHRA score III, n (%)*	395/2094 (18.9)	116/594 (19.5)	511/2688 (19.0)	
*EHRA score IV, n (%)*	39/2094 (1.9)	16/594 (2.7)	55/2688 (2.0)	
**ECG and echocardiogram characteristics**				
Bundle Branch Block, n (%)				0.17
*No*	1781/1983 (89.8)	490/562 (87.2)	2271/2545 (89.2)	
*LBBB*	111/1983 (5.6)	37/562 (6.6)	148/2545 (5.8)	
*RBBB*	91/1983 (4.6)	35/562 (6.2)	126/2545 (5.0)	
LVEF (%), median (IQR)	60 (55–65)	57 (50–62)	60 (53–65)	<0.001
LVEDD (mm), median (IQR)	51 (46–54)	50 (46–55)	50 (46–54)	0.89
LA size (AP diameter), median (IQR)				
*AP dimension, cm*	4.0 (3.7–4.5)	4.3 (3.9–4.7)	4.1 (3.8–4.5)	<0.001
*AP dimension index, cm/m*^2^	2.1 (1.9–2.3)	2.2 (1.9–2.5)	2.1 (1.9–2.4)	<0.001

AF = atrial fibrillation; AP= anterior-posterior; BMI = body mass index; CABG = coronary artery bypass grafting; CAD = coronary artery disease; CKD = chronic kidney disease; CMP = cardiomyopathy; COPD = chronic obstructive pulmonary disease; CV = cardiovascular; EHRA = European Heart Rate Association; DVT = deep vein thrombosis; CrCl C-G = creatinine clearance according to Cockroft-Gault formula; IQR, interquartile range; LBBB = left bundle branch block; LA = left atrium; LVEF, left ventricular ejection fraction; NYHA = New York Heart Association PCI = percutaneous coronary intervention; PAH= pulmonary arterial hypertension; PE = pulmonary embolism; RBBB = right bundle branch block; TE = thromboembolic; TIA = transient ischemic attack.

**Table 2:** Univariate and multivariable logistic regression analysis for factors associated with AF progression at 1 year of follow-up.

	Univariate Analysis			Multivariable Analysis		
	OR	95% CI	*p* Value	OR	95% CI	*p* Value
Age	1.02	1.01–1.02	<0.001	1.00	0.99–1.01	0.75
Female sex	0.86	0.72–1.04	0.12			
BMI	1.03	1.01–1.05	0.003	1.01	0.99–1.04	0.39
Hypertension	1.31	1.08–1.58	0.005	1.22	0.91–1.64	0.19
Diabetes mellitus	1.18	0.95–1.48	0.13			
Smoking	0.87	0.64–1.18	0.37			
No physical activity	1.34	1.09–1.64	0.004	1.35	1.02–1.78	0.03
Lipid disorder	0.97	0.81–1.18	0.82			
LVEF	0.98	0.97–0.98	<0.001	0.98	0.97–1.00	0.05
Dilated CMP	1.84	1.24–2.74	0.002	0.68	0.33–1.39	0.68
Hypertrophic CMP	2.01	1.21–3.34	0.007	1.40	0.65–3.02	0.38
PAH	1.48	0.93–2.36	0.09	0.72	0.36–1.44	0.36
Bundle branch block	1.18	0.85–1.62	0.31			
Left ventricular hypertrophy	1.34	1.07–1.68	0.01	1.01	0.74–1.37	0.94
Coronary artery disease	1.17	0.95–1.44	0.12			
Valvular disease	1.80	1.49–2.18	<0.001	1.63	1.23–2.15	0.001
Previous TE	1.21	0.91–1.60	0.18			
Previous haemorrhagic events	0.99	0.62–1.56	0.95			
Peripheral vascular disease	1.05	0.73–1.51	0.77			
Liver disease	0.99	0.50–1.94	0.97			
COPD	1.44	1.01–2.05	0.04	0.93	0.54–1.50	0.80
Dementia	1.25	0.48–3.17	0.64			
Anaemia	1.14	0.71–1.83	0.56			
Malignancy (prior or active)	1.17	0.83–1.66	0.35			
Hyperthyroidism	1.22	0.81–1.84	0.34			
Hypothyroidism	0.89	0.65–1.22	0.49			
CKD	1.18	0.87–1.60	0.28			
LA diameter	1.04	1.03–1.06	<0.001	1.03	1.01–1.05	0.005

CI = confidence interval; OR = odds ratio. For other abbreviations see Table 1.

**Table 3:** Rhythm-control interventions at baseline and association with AF progression at 1 year.

	No AF Progression (n = 2094, 77.9%)	AF Progression (n = 594, 22.1%)	Total (n = 2688)	*p*	OR [95% CI]	aOR [95% CI] Model 1	aOR [95% CI] Model 2
Rhythm-control interventions, n (%)	1279/2094 (61.1)	350/594 (58.9)	1629/2688 (60.6)	0.34	0.91 [0.76–1.10]	0.99 [0.82–1.20]	0.97 [0.81–1.17]
Rhythm-control type, n (%)				<0.001			
Only AADs	513/1279 (40.1)	101/350 (28.9)	614/1629 (37.7)		Ref	Ref	Ref
Electrical cardioversion	103/1279 (8.1)	65/350 (18.6)	168/1629 (10.3)		3.20 [2.19–4.67]	3.27 [2.24–4.77]	3.29 [2.25–4.81]
Pharmacological cardioversion	124/1279 (9.7)	41/350 (11.7)	165/1629 (10.1)		1.67 [1.11–2.53]	1.71 [1.12–2.58]	1.64 [1.08–2.48]
Catheter ablation	94/1279 (7.3)	8/350 (2.3)	102/1629 (6.3)		0.43 [0.20–0.92]	0.47 [0.22–1.00]	0.47 [0.22–1.02]
Mixed strategy	445/1279 (34.8)	135/350 (38.6)	580/1629 (35.6)		1.54 [1.15–2.05]	1.64 [1.22–2.19]	1.59 [1.19–2.12]

AADs, antiarrhythmic drugs; aOR, adjusted odds ratio, AF, atrial fibrillation, OR, odds ratio. Model 1 was adjusted analysis for age. Model 2 was adjusted analysis for CHA_2_DS_2_VASc score.

**Table 4:** Major adverse events at 2 years of follow-up according to AF progression status at 1 year.

	No AF Progression (n = 2094, 77.9%)	AF Progression (n = 594, 22.1%)	Total (n = 2688)	*p*	HR [95% CI]
All cause death, n (%)	54/2045 (2.6)	26/576 (4.5)	80/2621 (3.1)	0.02	1.78 [1.11–2.83]
MACE *, n (%)	58/1853 (3.1)	14/532 (2.6)	72/2385 (3.0)	0.55	0.88 [0.49–1.58]
CV death, n (%)	16/1942 (0.8)	7/560 (1.3)	23/2502 (0.9)	0.35	1.60 [0.65–3.89]
Any TE, n (%)	24/1917 (1.3)	5/549 (0.9)	29/2466 (1.2)	0.51	0.76 [0.29–2.01]
Stroke/TIA, n (%)	13/2030 (0.6)	2/569 (0.4)	15/2599 (0.6)	0.42	0.56 [0.12–2.52]
Any ACS, n (%)	26/1874 (1.4)	7/540 (1.3)	33/2414 (1.4)	0.87	0.98 [0.42–2.27]
Major bleeding, n (%)	11/2020 (0.5)	3/559 (0.5)	14/2579 (0.5)	0.98	1.00 [0.28–3.59]
Hospitalization for HF, n (%)	27/2008 (1.3)	12/548 (2.2)	39/2556 (1.5)	0.15	1.68 [0.85–3.32]

* MACE = composite of Any TE/ACS/CV death; HR, hazard ratio; CI confidence interval. For abbreviations see Table 1.

## Data Availability

Data available on request due to restrictions for privacy. Following a reasonable request, the corresponding author will consider to share some of the data.

## Supplementary Materials

The following supporting information can be downloaded at: https://www.mdpi.com/article/10.3390/jcm12030768/s1, Table S1: Pharmacological and antithrombotic management.
