# Supplementary material for: Factors Associated with Progression of Atrial Fibrillation and Impact on All-Cause Mortality in a Cohort of European Patients
Source: J Clin Med. 2023 Jan 18;12(3):768. doi: 10.3390/jcm12030768 (PMC9917523; doi:10.3390/jcm12030768)
Supplement: Supplementary file 1 [file jcm-12-00768-s001.zip › jcm-2121163-SI.pdf]

Supplementary Materials

Table S1. Pharmacological and antithrombotic management.

	**No AF Progression (n=2094, 77.9%)**	**AF Progression (n=594, 22.1%)**	**Total (n= 2688)**	**p**
**Pharmacological management,** n (%)				
Any antiarrhythmic treatment	944/1085 (45.3)	230/593 (38.8)	1174/2678 (43.8)	0.005
Amiodarone	461/2086 (22.1)	155/594 (26.1)	616/2680 (23.0)	0.04
Propafenone	197/2085 (9.4)	20/593 (3.4)	217/2678 (8.1)	<0.001
Flecainide	160/2085 (7.7)	27/584 (4.5)	187/2679 (7.0)	0.008
Sotalol	115/2085 (5.5)	25/594 (4.2)	140/2679 (5.2)	0.21
Beta-blockers	1371/2086 (65.7)	399/593 (67.3)	1770/2679 (66.1)	0.47
Digoxin	71/2085 (3.4)	54/593 (9.1)	125/2678 (4.7)	<0.001
ACE-inhibitors	810/2085 (38.8)	230/592 (38.9)	1040/2677 (38.8)	0.99
ARBs	396/2085 (19.0)	135/593 (22.8)	531/2678 (19.8)	0.04
Diuretics	695/2086 (33.3)	267/593 (45.0)	962/2679 (35.9)	<0.001
Aldosterone blockers	169/2085 (8.1)	76/592 (12.8)	245/2677 (9.2)	<0.001
Calcium channel blockers	416/2085 (20.0)	117/593 (19.7)	533/2678 (19.9)	0.90
Non-DHP - CCB	83/2085 (4.0)	28/593 (4.7)	111/2678 (4.1)	0.42
Statins	907/2085 (43.5)	258/591 (43.7)	1165/2676 (43.5)	0.94
Oral antidiabetics	284/2086 (13.6)	77/593 (13.0)	361/2679 (13.5)	0.69
Insulin	97/2085 (4.7)	25/593 (4.2)	122/2678 (4.6)	0.65
**Antithrombotic treatment**, n (%)				0.007
None	233/2092 (11.1)	41/593 (6.9)	274/2685 (10.2)	
Only antiplatelets	246/2092 (11.8)	57/593 (9.6)	303/2685 (11.3)	
VKA	650/2092 (31.1)	214/593 (36.1)	864/2685 (32.2)	
NOACs	737/2092 (35.2)	211/593 (35.6)	948/2685 (35.3)	
OAC plus antiplatelet	226/2092 (10.8)	70/593 (11.8)	296/2685 (11.0)	
Polypharmacy[#], n (%)	941/2078 (45.3)	305/588 (51.9)	1246/2666 (46.7)	0.005

ACE, angiotensin converting enzyme; AF= atrial fibrillation; ARB, angiotensin receptor blocker; CCB= calcium channel blockers, DHP, Dihydropyridine; NOAC= Non-Vitamin K Oral Anticoagulants; OAC= oral anticoagulants; VKA= Vitamin K antagonist. [#]Polypharmacy= contemporary use of five or more drugs.
